# 1212. COVID-19 and the Importance of Institutional Trust to Public Health

**DOI:** 10.1093/ofid/ofab466.1404

**Published:** 2021-12-04

**Authors:** Karin Kricorian, Katherine Kricorian

**Affiliations:** MiOra, Los Angeles, CA, Los Angeles, California

## Abstract

**Background:**

Institutional trust is a key component of the public health system’s effectiveness. However, the COVID-19 pandemic highlighted gaps in institutional trust and hesitation. Analysis was conducted to understand correlates of institutional trust to inform communication strategies for the ongoing pandemic and future public health crises.

**Methods:**

The Roper Center for Public Opinion/ America’s Voice Project Coronavirus Index conducted a US online survey February 22-April 5, 2021 and included questions about COVID-19 experiences, attitudes and behaviors. Respondents also indicated trust in each of four institutions to provide accurate information about COVID-19: federal government, state government, CDC and national public health officials. Scores were summed to create an Institutional Trust (IT) index: the top third was classified as “High IT,” the middle third “Moderate IT” and the bottom third “Low IT.” Data were analyzed using ***χ***2 tests, with z-tests for more granular between-group comparisons.

**Results:**

Those with Low IT were significantly more likely than those with Moderate or High IT to be white, male, rural, politically conservative, married, and live with children under age 18. Low IT individuals were less likely to have been tested for COVID-19 themselves and less likely to know someone who had tested positive for COVID-19. However, Low IT respondents were more likely to have tested positive for COVID-19, even when controlling for their lower propensity to be tested. Low IT individuals were significantly more likely to have visited restaurants and stores in the past week and feel these activities posed no health risk; they also wore masks less often. Despite greater risk-taking, Low IT respondents were over five times more likely than High IT respondents to refuse the COVID-19 vaccine.

**Conclusion:**

Low IT was associated with higher COVID-19 vaccine hesitancy as well as behavior that, at the time data was collected, put people at higher risk of contracting COVID-19. Public health officials should prioritize the development of more effective communications towards Low IT populations. Traditional methods of establishing message credibility may require modification in order to encourage Low IT individuals to participate in behaviors that enhance public health.

**Disclosures:**

**All Authors**: No reported disclosures

